# A Survey Study of the Workplace Experiences of Young Adult Peer Support Workers in the United States

**DOI:** 10.1007/s10597-025-01585-3

**Published:** 2026-01-07

**Authors:** Haley Payne, Dionne Regis, Margaret Smith, Rashni Stanford, Brandon Bond, Elizabeth Thomas

**Affiliations:** 1 School of Professional & Applied Psychology, Philadelphia College of Osteopathic Medicine, Philadelphia, United States; 2 Department of Psychology, University of North Texas, Denton, United States; 3 College of Public Health, Temple University, Philadelphia, United States; 4 Deep Space Mind 215, Philadelphia, United States; 5 College of Literature, Science, and the Arts, University of Michigan–Ann Arbor, Ann Arbor, United States

**Keywords:** Peer specialists, Youth, Mental health, Workplace satisfaction

## Abstract

This study examined the workplace experiences of young adult peer support workers (YPSWs), including how these experiences differ by certification status and what barriers and recommendations YPSWs identify for improving their work environments. A cross-sectional survey was conducted via REDCap with 49 YPSWs aged 18–30 currently employed in a paid peer support role in the United States. Participants completed quantitative items assessing satisfaction with various workplace aspects, supervision, intergenerational inclusiveness, and stigma, and open-ended questions about workplace barriers and recommendations. Analyses included descriptive statistics, one-way ANOVA tests comparing certified and non-certified YPSWs, and thematic analysis of qualitative responses. Respondents reported relatively high satisfaction with fringe benefits, training, and supervision, but lower satisfaction with opportunities and rewards. Most reported a positive intergenerational climate and little stigma from coworkers. Youth certified peer specialists reported significantly lower satisfaction with opportunities and rewards and benefits than non-certified peers, and significantly lower satisfaction with supervision than general certified peer specialists. Qualitative data revealed key workload, policy, and individual barriers as well as age-related issues (e.g., lack of respect, imposter syndrome). Respondents recommended improvements for leadership, policy, job incentives, workforce capacity and support, peer integration, and collaboration and inclusion. Findings highlight both strengths and challenges in YPSW work environments, with notable disparities by certification status. Given their unique developmental stage and experiences of marginalization, strengthening this workforce will require attention to compensation, role clarity, and inclusion through a positive youth development lens.

## Introduction

Young adult peer support workers (YSPWs) have become an increasingly important segment of the peer support workforce, playing a critical role in addressing the youth mental health crisis in the United States (Department of Health and Human Services, 2021). Young adult peer support occurs when young persons (typically aged 18–30) who have lived experience of a mental health condition work with near-aged individuals to share their experience, strength, and hope (Gopalan et al., 2017; see below for state certification age requirements). YPSWs can contribute to mental health service settings by facilitating engagement, providing emotional support, assisting with navigation and planning, advocating for service users, and contributing to research and education efforts (de Beer et al., 2022). YPSWs have been effective in engaging young people hesitant to seek mental health services (Ojeda et al., 2021), and can help reduce stigma by reframing lived experience as a source of strength rather than a deficit (Halsall et al., 2023).

The benefits of young adult peer support also apply to YPSWs themselves. For young adults with mental health conditions, employment may lead to enhanced self-esteem and better management of mental health problems (Torres Stone et al., 2018). Involvement in peer support roles helps YPSWs increase self-acceptance, improve social and professional skills, and enhance career opportunities (Halsall et al., 2023; Simmons et al., 2020). Workplace factors particularly related to YPSW satisfaction and success include access to supportive supervision and a larger peer support community, open communication with non-peer providers, and increased job autonomy, rewards, and recognition (Delman & Klodnick, 2017; Singh et al., 2025; Tisdale et al., 2021).

Despite its promise, young adult peer support remains a developing field with significant challenges. The infrastructure to support YPSWs is still limited, and, similar to reports from the adult peer support literature (Ostrow et al., 2025; Scanlan et al., 2020), many programs lack clear role definitions, structured supervision, and adequate compensation (Halsall et al., 2023; Singh et al., 2025; Tisdale et al., 2021). Moreover, stigma and differential treatment in workplace settings remain key barriers to fully integrating YPSWs into mental health teams (de Beer et al., 2022), reflecting broader patterns across the peer workforce (Abraham et al., 2021; Scanlan et al., 2020). For YPSWs, these challenges may reflect intersecting biases related to both mental health and age. For example, Firmin et al. (2019) found that peer supporters face microaggressions tied to their mental health and their roles. YPSWs also report experiencing adultism – prejudice against young people (LeFrançois, 2014) – in the workplace (de Beer et al., 2022; Delman & Klodnick, 2017), which has been linked to decreased organizational commitment, lower job satisfaction, and increased turnover intentions (Schmitz et al., 2025). YPSWs also have unique developmental needs, as this role is often one of their first forays into the workplace and they may need time to hone professional and life skills (Delman & Klodnick, 2017; Hermsen-Kritz, 2020). Currently, it is unclear the degree to which these needs are addressed through training and supervision (Gopalan et al., 2017).

To strengthen workplace supports for YPSWs, it is crucial to gain deeper insight – directly from YPSWs – about their job experiences, including sources of satisfaction and dissatisfaction, challenges, and effective solutions. While some research has explored YPSWs’ experiences, it has often relied on qualitative methods and small samples within specific workplace settings (Delman & Klodnick, 2017; Halsall et al., 2023; Singh et al., 2025; Tisdale et al., 2021). Additionally, the field lacks a nuanced understanding of how workplace experiences may differ based on YPSW characteristics. For example, examining differences based on certification status could provide valuable insights. At least 49 states and the District of Columbia offer “general” peer certification programs with at least 5 (i.e., Kentucky, Georgia, Ohio, New York, Tennessee) developing more specialized youth certified peer specialist trainings (University of Illinois-Chicago Center on Integrated Health Care & Self-Directed Recovery, n.d.). Based on a review of state websites, these programs vary in eligibility criteria – particularly in age range – with Georgia defining eligible individuals as 18–26 years, New York, Ohio, and Tennessee as 18–30 years, and Kentucky as 18–35 years. All require lived experience with behavioral health or related challenges, prior service use in youth-serving systems, and completion of a minimum number of training hours (15–40). However, the specific content and competencies addressed in youth versus general peer specialist trainings remain largely undocumented (Gopalan et al., 2017), limiting understanding of how differences in preparation may shape workplace experiences. It is also possible that, regardless of type, holding a certification may influence YPSWs’ roles, expectations, credibility, and access to resources, potentially shaping their experiences in distinct ways from non-certified counterparts. Understanding these nuances can help inform more tailored and impactful workplace supports.

The purpose of this study was to conduct a survey of YPSWs in the United States, including those with general or youth-specific certification as well as non-certified individuals, to better understand their workplace experiences. As such, the study addressed three primary research questions:

1. What are the workplace experiences of YPSWs?
2. How do workplace experiences differ based on certification status?
3. What do YPSWs perceive as barriers to their success in the workplace? What recommendations do they have for how their workplaces can be improved?

## Methods

### Participants

This study utilized a cross-sectional survey to gather data from YPSWs within the United States. Survey respondents were recruited online through study flyers containing a link to the survey, which were distributed via email listservs of national peer support organizations (e.g., National Association of Peer Supporters) as well as 35 statewide networks (e.g., Pennsylvania Peer Support Coalition). Eligibility criteria required respondents to be 18–30 years old, currently employed full-time or part-time in a paid peer support role, fluent in English, and able to provide informed consent. We included YPSWs aged 18–30 to align with the most common age range used across state certification programs. The survey was open to individuals who received peer specialist certification (general and/or youth-specific) and to non-certified peer support workers.

Data collection was conducted using REDCap (Research Electronic Data Capture) (Harris et al., 2009, 2019) between March 2023 and April 2024. Respondents were provided detailed study information and required to indicate informed consent before accessing the survey, which took approximately 30 min to complete. To encourage participation, respondents could enter a raffle to win an electronic gift card: one valued at $100 and two at $50. The study was approved by the Institutional Review Board at the senior author’s institution.

## Measures

Survey items were derived from validated measures of workplace experiences in the general population or were developed for this study, with input from a working group that included YPSWs and experts in young adult mental health services. Demographic items included race/ethnicity and gender identity; peer support experience covered certification and work settings. Workplace experiences addressed satisfaction with pay, benefits, training, and supervision, and perceptions of workplace intergenerational climate and stigma. Open-ended questions allowed respondents to elaborate on workplace barriers and suggest changes or improvements they believed were necessary.

Items on pay, benefits, and training came from the Job Training and Job Satisfaction Survey (Schmidt, 2004), measuring attitudes toward job conditions across settings. The survey included items from the Opportunities and Rewards, Fringe Benefits, Organizational Support for Training, and Employee Satisfaction with Training Items subscales, rated on a six-point Likert Scale (1=“Disagree Very Much” to 6=“Agree Very Much”); higher scores indicate greater satisfaction. The original scale has demonstrated construct validity and adequate reliability (α = 0.83–0.89) (Schmidt, 2004); reliability in this study ranged from α = 0.73–0.92.

The Satisfaction with Supervision Scale (Noelker et al., 2009) was used to assess satisfaction with various aspects of the supervisory relationship including communication, feedback, recognition, and support. Items are rated on a 3-point scale (0=“Hardly Ever” to 2=“Most of the Time”); higher scores indicate greater satisfaction. Two items not relevant to peer support were removed, and several were adapted for appropriateness. Previous research has shown the scale to have acceptable reliability (α = 0.94) and construct validity (Noelker et al., 2009). Cronbach’s alpha in the study sample was 0.92.

Workplace intergenerational climate was assessed using the Workplace Intergenerational Climate Scale (King & Bryant, 2017), measuring the presence of ageism in the workplace. Four items were derived from the Workplace Generational Inclusiveness subscale, two from the Workplace Intergenerational Retention subscale, and one original item was added (“In my workplace, being younger is viewed as a strength”). Items are rated on a 1 (“Strongly Disagree”) to 4 (“Strongly Agree”) scale, with higher scores indicating more positive perceptions. The measure exhibits strong content, structural, convergent, and discriminant validity, along with acceptable reliability (King & Bryant, 2017). In this study, only the Workplace Generational Inclusiveness subscale demonstrated acceptable reliability (α = 0.78) and was included in analyses for research question 2.

Mental illness stigma was assessed using the Stigma Scale (King et al., 2007). Selected items from the measure’s three subscales – Disclosure, Discrimination, and Positive Aspects – were included in the survey. Items were adapted to reflect perceived stigma from coworkers (e.g., “Other people…” was changed to “My coworkers…”). Items are rated on a scale of 0 (“Strongly Disagree”) to 4 (“Strongly Agree”); higher scores indicate greater perceived stigma. The scale demonstrates adequate internal consistency (α = 0.87) and evidence of concurrent validity (King et al., 2007). In this study, Disclosure, Discrimination, and total scores showed acceptable reliability (α = 0.81–0.90) and were included in analyses for research question 2.

### Data Analysis

Quantitative analyses were conducted using SPSS version 30. To address research question 1 about YPSWs’ experiences in the workplace, descriptive statistics (i.e., counts, frequencies) were reported by item. Whenever possible, response categories were combined into “disagree” and “agree” to enhance the interpretability of each item response.

To address Research Question 2 regarding differences in workplace experiences by certification status, a series of one-way ANOVA tests were conducted. Relevant subscale and total scores from each measure served as the dependent variables, while certification status – categorized as general certified peer specialist only (1 = yes, 0 = no), youth certified peer specialist (including those with youth certification only or in addition to general certification) (1 = yes, 0 = no), and non-certified (1 = yes, 0 = no) – was used as the independent variable. Prior to analysis, relevant items were reversed to ensure consistent scaling. Homogeneity of variances was assessed using Levene’s test. Tukey’s HSD was used for post hoc comparisons when variances were equal, and Games–Howell tests were used when they were not.

To address research question 3 related to workplace barriers and recommendations, open ended items were analyzed qualitatively by two independent coders using integrated thematic analysis (Bradley et al., 2007). The first and third authors reviewed and annotated each open-ended response, noting barriers and recommendations discussed by each respondent. Based on these notes, they developed a coding template with three categories: barriers, age-related issues, and recommendations. They independently coded all open-ended responses and resolved discrepancies through discussion.

## Results

### Respondent Characteristics

A total of 95 responses were collected. After thorough data cleaning, 13 entries were excluded because respondents indicated “no” to the eligibility confirmation item and did not complete the survey, 31 were removed for not answering any survey questions, and 2 were identified as duplicates. This process resulted in a final sample of 49 respondents.

As shown in Table 1, most respondents were White and female, with a smaller proportion identifying as male or as members of racial, ethnic, and gender minoritized groups. Most reported having psychiatric diagnoses of anxiety or mood disorder; other diagnoses included schizophrenia or schizoaffective disorder, personality disorder, or “other.” The majority reported an annual salary range between $20,000-$49,000. The most frequently reported work settings were outpatient and tele-mental health; with some reporting that they worked in “other” settings, including schools, crisis centers, and non-profit organizations. Seventeen reported being certified general peer specialists only, 4 reported being certified youth peer specialists only, 13 were dually certified with general and youth certifications, and 15 were non-certified. Individuals reported being certified in the following states: California, Delaware, Florida, Georgia, Kentucky, Maine, Massachusetts, Michigan, Missouri, New York, Ohio, Pennsylvania, Tennessee, and Virginia. On average, respondents were approximately 26 years of age.

**Table 1:** Respondent characteristics

	*N*	%
Race/ethnicity^a^		
Asian	3	6
Black or African American	10	20
White	36	74
Hispanic or Latino	8	16
Other	1	2
Gender identity		
Male	5	10
Female	36	74
Transgender	1	2
Non-binary/conforming	6	12
Other	1	2
Psychiatric Diagnosis^a^		
Anxiety Disorder	41	84
Schizophrenia or Schizoaffective Disorder	2	4
Mood Disorder	31	63
Personality Disorder	7	14
Other	10	20
Annual Salary Range		
Under $10,000	2	4
$10,000 - $19,000	6	12
$20,000 - $49,000	36	74
$50,000 - $99,000	5	10
$100,000 or higher	0	0
Work Setting(s)^a^		
Inpatient	4	8
Outpatient	27	55
Consumer-run program	13	27
Residential	9	18
Tele-mental health (e.g., telephone, videoconference)	18	37
Virtual (e.g., social media, gaming, streaming platform)	11	22
Other	17	35
Certification Status		
Certified General Peer Specialist Only	17	35
Certified Youth Peer Specialist Only	4	8
Certified General and Youth Peer Specialist	13	27
Non-Certified	15	31
Length of Time Worked In Peer Support Field		
Less than 1 year	10	20
1–2.99.99 years	27	55
3–4.99.99 years	8	16
5 years or greater	3	6
	M	SD
Age	25.89	3.05

^a ^Categories were not mutually exclusive; ^b ^*N*=48

### Workplace Experiences

As shown in Table 2, most respondents reported low satisfaction related to pay – only 21 (43%) agreed to some extent that they were paid fairly, and 28 (57%) expressed some level of agreement that they felt underappreciated based on their compensation. Conversely, respondents reported relatively high satisfaction with fringe benefits, organizational support for training, and training experiences: 37 (76%) agreed to some extent that benefits were competitive, 40 (82%) felt training was encouraged, and 45 (92%) reported being able to apply training to their work. Most respondents perceived a positive intergenerational climate, with 38 (78%) agreeing to some extent that all ages are respected, though fewer agreed that being younger is seen as a strength.

**Table 2:** Workplace experiences – job training and job satisfaction scale (*N* = 49) and workplace intergenerational climate scale (*N* = 46) (Item scores)

Item	Disagree		Agree	
	*N*	%	*N*	%
Job Training and Job Satisfaction Scale - Opportunities and Rewards				
I feel I am being paid a fair amount for the work I do.	28	57	21	43
Raises are too few and far between.	9	18	39	80
I feel unappreciated by the organization when I think about what they pay me.	21	43	28	57
I feel satisfied with my chances for salary increases.^a^	31	63	17	35
Job Training and Job Satisfaction Scale - Fringe Benefits				
I am not satisfied with the benefits I receive.	30	61	19	39
The benefits we receive are as good as most other organizations offer.	12	24	37	76
There are benefits we do not have which we should have.	25	51	24	49
Job Training and Job Satisfaction Scale - Organizational Support for Training				
My department provides learning/training opportunities to meet the changing needs of my workplace.	8	16	41	84
In my department, learning is planned and purposeful rather than accidental.	12	24	37	76
In my department, people are interested in both personal and professional development.	5	10	44	90
Training and development are encouraged and rewarded in my department.	9	18	40	82
Job Training and Job Satisfaction Scale - Employee Satisfaction with Training				
Overall, the on-the-job training I receive is applicable to my job.	7	14	42	86
Overall, the training I receive on the job meets my needs.	6	12	43	88
Overall, I am satisfied with the amount of training I receive on the job.^a^	13	27	35	71
I am generally able to use what I learn in on-the-job training in my job.^a^	3	6	45	92
Workplace Intergenerational Climate Scale				
I believe that my work environment is a healthy one for people of all ages.	11	22	35	71
Workers of all ages are respected in my workplace.	8	16	38	78
I am able to communicate effectively with workers of different generations.	6	12	40	82
Working with co-workers of different ages enhances the quality of my work.	3	6	43	88
I feel pressure from older workers to step down.	38	76	8	16
In my workplace, qualified younger workers tend to be overlooked for promotions.	27	55	19	39
In my workplace, being younger is viewed as a strength.^b^	26	53	19	39

^a^ *N*=48; ^b^ *N*=45

As shown in Table 3, respondents reported high satisfaction with supervision, with at least 60% indicating their supervisors regularly demonstrated key supportive behaviors (e.g., active listening, recognition, constructive feedback, and respect). Most disagreed their supervisors dismissed their input or acted superior. Responses were more mixed on supervisors’ availability, fairness in delegation and conflict resolution, and handling of underperformance.

**Table 3:** Workplace experiences – satisfaction with supervision scale (Item scores) (*N* = 45)

Item	Hardly Ever		Some of the Time		Most of the Time	
	*N*	%	*N*	%	*N*	%
Listens carefully to my observations and opinions	1	2	11	22	33	67
Gives me credit for my contributions to client care	2	4	9	18	34	69
Lets me know how helpful my perspectives are for supporting people	4	8	9	18	32	65
Shows me recognition when I do good work	3	6	12	25	30	61
Helps with work when help is needed	2	4	14	29	29	59
Takes responsibility for her/his mistakes	4	8	12	25	29	59
Encourages me to use my peer support skills to the fullest	0	0	11	22	34	69
Disciplines or removes staff who do not do their job well	13	27	15	31	17	35
Is unavailable when I need her/him	22	45	20	41	3	6
Understand how hard I work	3	6	11	22	31	63
Delegates tasks in a fair manner	6	12	15	31	24	49
Is fair when handling conflicts	5	10	15	31	25	51
Treats me as an equal member of the support team	2	4	9	18	34	69
Ignores my input when developing support plans	34	69	8	16	3	6
Acts like he or she is better than I am	36	74	4	8	5	10

Table 4 shows that many respondents did not endorse experiencing stigma from coworkers: 27 (55%) disagreed they had been discriminated against due to mental health, and 22 (45%) agreed they felt comfortable with coworkers knowing about their mental health history.

**Table 4:** Workplace experiences – stigma scale (Item scores) (*N* = 29)

Item	Disagree		Neither Agree nor Disagree		Agree	
	*N*	%	*N*	%	*N*	%
Discrimination						
I have been discriminated against by my coworkers because of my mental health problems.	27	55	1	2	1	2
Sometimes I feel that I am being talked down to by my coworkers because of my mental health problems.	23	47	2	4	4	8
I have been discriminated against by employers because of my mental health problems.	22	45	2	4	5	10
I have not had any trouble from my coworkers because of my mental health problems.^a^	4	8	4	8	20	41
My coworkers have avoided me because of my mental health problems.	24	49	5	10	0	0
My coworkers have insulted me because of my mental health problems.^a^	25	51	3	6	0	0
Disclosure						
I do not mind my coworkers knowing I have had mental health problems.	5	10	2	4	22	45
I worry about telling my coworkers that I receive mental health treatment.	21	43	7	14	1	2
My coworkers reactions to my mental health problems make me keep to myself.	21	43	6	12	2	4
I do not feel embarrassed around my coworkers because of my mental health problems.^a^	4	8	3	6	21	43
I avoid telling my coworkers about my mental health problems.	15	31	8	16	6	12

^a^ *N*=28

### Comparisons Based on Certification Status

As shown in Table 5, there were significant differences by certification status related to satisfaction with opportunities and rewards, fringe benefits, and supervision. Tukey’s HSD test for multiple comparisons revealed that group differences on the Job Satisfaction and Job Training Scale were between those in the youth certified peer specialist group and the non-certified group (opportunities and rewards: *p* <.01, 95% CI = [−10.37, −2.00]; fringe benefits: *p* <.05, 95% CI = [−6.04, −0.13]), with youth certified peer specialists having significantly lower satisfaction in these areas. The Games-Howell test indicated that youth certified peer specialists were also significantly less satisfied with supervision than general certified peer specialists (*p* <.05, 95% CI = [−12.01, −0.65]). There were no significant differences between groups related to organizational support for training, satisfaction with training, perceptions about intergenerational inclusiveness, or stigma beliefs.

**Table 5:** Workplace experiences of young adult peer support workers (Scale scores and comparisons)

Scale	Overall Sample (*N* = 49)		General Certified Peer Specialist Only (*n* = 17)		Youth Certified Peer Specialist (*n* = 17)		Non-Certified (*n* = 15)				
	M	SD	M	SD	M	SD	M	SD	F	df	*p*
Job Training and Job Satisfaction Survey											
Opportunities and Rewards^a^	11.51	5.25	11.18	5.33	8.81	4.04	15.00	4.64	6.49	2,44	< 0.01^**^
Fringe Benefits	11.41	3.62	12.00	3.00	9.65	3.71	12.73	3.59	3.59	2,46	0.04^*^
Organizational Support for Training	18.35	4.15	19.47	3.54	17.29	4.27	18.27	4.57	1.18	2,46	0.32
Employee Satisfaction with Training^b^	18.48	4.54	18.94	4.02	17.94	5.07	18.53	4.76	0.20	2,45	0.82
Total Score^a^	63.81	13.64	66.47	11.23	57.31	14.28	68.00	13.71	3.05	2,44	0.06
Satisfaction with Supervision Scale											
Total Score^c^	23.62	6.47	26.06	3.42	19.73	7.99	25.00	5.71	4.91	2,42	0.01^*^
Workplace Intergenerational Climate Scale											
Workplace Generational Inclusiveness^d^	12.63	2.37	13.41	2.35	11.47	2.67	12.93	1.59	3.11	2,43	0.06
Stigma Scale											
Disclosure^e^	5.07	4.61	3.70	4.55	6.13	5.11	5.60	4.40	0.70	2,25	0.51
Discrimination^f^	4.26	4.39	2.50	3.17	6.50	5.73	4.22	3.70	1.99	2,24	0.16
Total Score^g^	10.35	9.34	6.80	7.63	14.00	12.64	11.44	7.67	1.36	2,23	0.28

^a^ *N*=47; ^b^ *N*=48; ^c^ *N*=45; ^d^ *N*=46; ^e^*N*=28; ^f^*N*=27; ^g^*N*=26; ^*^ *p* <.05; ^**^ *p* <.01

### Barriers

Qualitative analyses revealed several workplace barriers and recommendations (Table 6). While 9 respondents reported having no workplace barriers, 18 reported barriers in the following areas: workload barriers, policy barriers, individual barriers. Twenty-two did not answer the question about barriers.

**Table 6:** Barriers and recommendations

Theme	*N*	%	Exemplar Quotes
Barrier Themes	27		
Workload Barriers			
Limited Time	7	26	“Limited time for the classes I facilitate, too much material to cover in one class period sometimes, and not enough time to build relationship with peers over the course.” −154
Caseload Issues	5	19	“Working way over capacity (large caseload and limited time) creates stress and prevents connecting with my peers in certain occasions.” −187
Policy Barriers			
Policy Restrictions Interfering with Ability to Support Peers	8	30	“Policies prohibiting the use of social media to communicate with peers.” −192
Individual Barriers			
Personal Barriers	1	4	“I also face a lot of personal barriers that prevent me from best supporting my peers such as financial barriers or mental health barriers.” −171
Difficulty Getting Certified	1	4	“Getting peer certification shouldn’t take long to get and having people pay out of pocket to get the training.” −166
Age-Related Issues	20		
Lack of Respect	14	70	“I’m often over-looked, spoke over, made to feel like I don’t know what I’m doing and it can be difficult.” −138
Have to Work Harder Due to Age	3	15	“I am the youngest at my job and I do 3 times the amount of work and will be given tasks that do not apply to my role but the older staff do not want to do it themselves.” −197
Difficulty Connecting with Coworkers	2	10	“My communication style is different than my counterparts of different ages…” −140
Imposter Syndrome	2	10	“Sometimes I feel that I do not have enough experience to be in the role I am in.” −167
Not Provided Adequate Benefits	2	10	“Our pay rate is hourly compared to our other older co-workers who are salary. We struggle financially because we use our personal cars to transport, our benefits are really expensive, and this organization just doesn’t really take into consideration what it is like to be a young person in today’s world and the barriers we face.” −171
Training does not Focus on Young Adults	1	5	“Often folks will say they want our perspective as young folks, but there is no State certification in youth peer support, and our intentional peer support training does not cover youth and young adults, it is very older adult focused. So the challenges we have are often not covered or taught, and there is often a lot of youthism in the trainings we take, so we have to almost untrain ourselves while we are training on things, depending on where the training comes from.” −140
Recommendation Themes	46		
Leadership Recommendations			
More Involvement from Leadership	6	13	“People in leadership positions standing up to those who are seasoned and let them know times have changed and redirecting them for our young adult expertise rather than assuming they know best for the “kid” or “child"” −138
Leadership Listening to Suggestions	5	11	“Consider suggestions more often.” −156
Policy Recommendations			
Enhancing Structure through Policy	3	7	“Providing more of a structure[d] work environment.” −114
Job Incentive Recommendations			
Increasing Pay	13	28	“Hire more peers and pay them better so they stay longer, offer better long-term pay raises and career opportunities rather than pushing for peers to be promoted into clinical roles instead of remaining as peers.” −113
Increasing Benefits	3	7	“Providing no-questions-asked PTO for mental health days/unlimited PTO, ensuring that pay is equal to the cost of living in the area.” −168
Offering Opportunities for Growth	7	15	“Give promotions/raises for extra efforts such as peer specialist certification, and for years of experience.” −165
Workforce Capacity and Support Recommendations			
Offering Ongoing Training	14	30	“Continuing intentional monthly staff meetings with training/skill check ins.” −195
Providing Resources	5	11	“A transport car so we don’t have to use our own car and gas” −171
Hiring More Staff	7	15	“Hiring more peers to help with the amount of people we support.” − 119
Adjusting Workload/Type of Work	6	13	“I work a lot with the county contract and my time is not always respected. I think there needs to be more formal communication from upper management that although we hold your contract does not [mean] my job is to solely tend to your needs. This job is a lot of administration work and not much peer support work. I have worked here almost a year and I have not worked with a single young person. This either needs to be a community liaison role or a peer role. There is only so much of me to go around and I can’t do everything.” −111
Peer Integration Recommendations			
Offering Opportunities for Peers to Contribute	13	28	“Offer a spotlight on the peer voice at the weekly meeting.” −119
Valuing Lived Experience	9	20	“I think they could emphasize how important having the lived experience is and what a vital role we play as peer supports so the rest of the company doesn’t look at us like uneducated or too young.” −171
Collaboration and Inclusion Recommendations			
Promoting Diversity	6	13	“Work with more diverse groups within the community” −142
Improving Team-Based Communication	11	24	“I feel like more communication would help and if everyone was on the same page…” −169
Engagement with Other Programs/Support Systems	2	4	“A separate but connected adult or family peer support team so that guardians and parents of the peers we work with have someone to talk to, get help, or ask questions. This responsibility often falls on us because the adult in our peers’ life is often also needing a lot of support and we are unable to provide it in order to do peer support for the young folks.” −140

Respondents commonly reported having limited time for their work with peers, with many attributing this problem to having large caseloads. Organizational policies, such as restricting social media communication with peers, contributed significantly to their quality of work. There was additional discussion about the lack of a clear structure for peer work, which was attributed to the absence of formal policies guiding this area. Other barriers related to personal barriers (e.g., mental health challenges), and certification difficulties.

### Age-Related Issues

Twenty respondents described age-related issues. Many expressed feelings of not being respected, having to work harder due to their age, and a general sense of imposter syndrome. Others reported that they had difficulty connecting with coworkers because of generational differences, such as differences in communication styles. Other age-related issues included inequitable salary and benefits, being assigned tasks outside of the job description – often because older workers were unwilling to do them – and organizational training that failed to address key topics necessary for effectively working with youth.

### Recommendations

While one respondent indicated that they had no recommendations, 46 provided recommendations in the following areas: leadership, policy, job incentives, workforce capacity and support, peer integration, and collaboration and inclusion. Many highlighted the need for greater leadership engagement, including increased leadership involvement in addressing age-related dynamics and more openness to peer feedback. Several recommendations focused on policy improvements, such as establishing clearer structures and expectations to guide daily work. Respondents also emphasized the importance of job incentives, calling for higher pay, improved benefits, and expanded opportunities for advancement. Strengthening workforce capacity and support was another common subtheme, with suggestions for ongoing training, additional resources, hiring more staff, and adjusting workloads to better align with peer support duties. Recommendations related to peer integration underscored the value of elevating lived experience and creating more opportunities for YPSWs to meaningfully contribute within their teams. Finally, respondents advocated for efforts to enhance collaboration and inclusion, including promoting diversity, improving team-based communication, and fostering stronger connections with other programs or support systems.

## Discussion

To our knowledge, this was the first survey of YPSWs across diverse peer support roles in the United States, expanding upon prior smaller, primarily qualitative studies in this area. Findings highlight key areas of relative job satisfaction and dissatisfaction, offering insight into how these experiences differ based on certification status. Additionally, the study contributes to the expanding body of research on perceived workplace barriers and strategies for addressing them within this unique workforce. By centering the voices of YPSWs, it provides critical guidance for improving workplace conditions, supporting professional development, and ultimately strengthening the peer support workforce that plays a vital role in youth mental health services.

Findings affirm and expand prior studies on the workplace experiences of YPSWs. In alignment with prior qualitative research (Singh et al., 2025), a significant proportion of respondents reported dissatisfaction with their pay, highlighting an ongoing concern about the adequacy of compensation in this workforce. Simultaneously, respondents expressed relatively high levels of satisfaction with other aspects of the workplace, such as fringe benefits, training, and supervision. These findings are encouraging, especially in light of previous research demonstrating the importance of “organizational social capital” – encompassing factors such as supportive supervision and access to initial and ongoing trainings – as key facilitators of YPSWs’ job success (Delman & Klodnick, 2017). Quantitative findings diverge from previous research regarding stigma and adultism (de Beer et al., 2022; Delman & Klodnick, 2017), likely due to methodological differences (e.g., structured scales vs. open-ended interviews). Despite lower ratings on ageism-related items, many respondents still described experiencing age-related workplace barriers.

A particularly novel and important finding of this study is that several aspects of workplace satisfaction were lower among those certified as youth peer specialists compared to general certified or non-certified peers. Compared to general certified peers, youth certified peers were less satisfied with their supervision experiences, and compared to non-certified peers, youth certified peers were less satisfied with their pay and benefits. Several factors may explain these discrepancies. Youth-specific certification may unintentionally emphasize a worker’s age in ways that contribute to feelings of marginalization. Youth certified peers may also anticipate greater support or recognition as a result of completing a specialized certification, creating a mismatch between expectations and the realities of their workplace conditions. These findings point to a need to strengthen workplace conditions to ensure that youth certified peer specialists are adequately recognized for their training and commitment and are adequately supported so that their roles remain sustainable.

In alignment with other qualitative findings, YPSWs in this study cited challenges with respect from non-peer colleagues, unclear role expectations, and limited access to youth-specific training (Delman & Klodnick, 2017; Singh et al., 2025; Tisdale et al., 2021). They also highlighted less commonly reported barriers such as large caseloads, time constraints, restrictive policies, and being assigned tasks undesirable to older workers. Certain recommendations were consistent with past studies, including the need for improved communication processes with colleagues and workplace policies that bring more structure and clarity to the YPSW role (Delman & Klodnick, 2017; Singh et al., 2025). A novel suggestion included diversifying the peer workforce (e.g., hiring family peers) to avoid YPSWs serving in dual roles supporting both youth and their parents.

### Implications

Given their important role in addressing workforce shortages and engaging hard-to-reach youth amid the United States’ youth mental health crisis, as well as their unique needs and experiences, special attention to the retention of YPSWs is warranted. Organizations should prioritize equitable compensation, for example, by advocating for Medicaid reimbursement for YPSW services separately from adult and family peer services – since many adult models require prior work experience or education that could exclude capable YPSWs (Gopalan et al., 2017) – and by establishing policies linking wages to certification and tenure (Delman & Klodnick, 2017). Addressing structural barriers like unclear roles, high caseloads, and inconsistent expectations across systems is critical (Tisdale et al., 2021). Organizational culture must also confront adultism by educating staff on the value of lived experience and integrating YPSWs as full team members through meaningful roles and formal recognition (Delman & Klodnick, 2017; Tisdale et al., 2021). Finally, professional development for this role would benefit from an infusion of positive youth development principles that account for the unique developmental needs of young adults (Donlan et al., 2015), as well as the barriers YPSWs may face personally as they move through their own recovery journeys. This would include a focus on skill-building, opportunities for leadership, community involvement, building confidence, and caring supervision and mentorship for YPSWs from professionals who understand the unique intersection of experiences within this role (Donlan et al., 2015; de Beer et al., 2022; Hermsen-Kritz, 2020; Tisdale et al., 2021).

There is also a pressing need for research examining disparities in workplace experiences among YPSWs, especially the factors that shape these experiences. Exploratory analyses conducted as part of this study yielded no clear pattern of differences in satisfaction by workplace setting. However, qualitative recommendations indicate that organizational conditions, such as leadership practices, role clarity, workforce support, and peer inclusion, may play an important role in shaping YPSWs’ workplace experiences. Future studies should also investigate how certification impacts perceived legitimacy, role clarity, compensation, and job satisfaction. Moreover, research is needed to evaluate the adequacy of YPSW training in addressing their unique needs and the long-term outcomes of workforce development programs (Gopalan et al., 2017). Finally, future research should examine YPSWs’ workplace well-being, focusing on how organizational culture, supervision quality, and boundary-setting practices influence burnout and retention (Tisdale et al., 2021).

### Limitations

Several limitations should be noted. The sample, primarily Caucasian females, may not be representative of the broader YPSW workforce. The total sample size (49 respondents) was modest and recruitment was challenged by the lack of centralized registries for this subgroup. Further, a response rate could not be calculated due to the use of network-based recruitment strategies and the absence of workforce estimates for YPSWs aged 18–30 years. While this limited our ability to assess survey reach, this approach was necessary to engage a geographically diverse and otherwise difficult-to-identify workforce. Future research is needed to obtain an accurate estimate of the number of individuals under the age of 30 working in YPSW roles. There was also a substantial amount of missing data on the Stigma Scale, which was added after data collection had begun; thus, related findings should be interpreted with caution. With only four open-ended questions and low response rates for some of these items, the study offers limited qualitative depth regarding workplace barriers and recommendations and may not capture the full range of YPSW experiences. Finally, some relevant variables, including training experiences and certification eligibility, were not collected in this study and warrant examination in future research. This is particularly important given that respondents from some states did not have the opportunity to obtain youth peer specialist certification because such programs were not available in their states. Despite these limitations, this study contributes important preliminary data to an underdeveloped area of research. The findings provide valuable early insight into the workplace experiences of a small but growing segment of the peer support workforce and offer a foundation for future research to build upon with larger and more diverse samples.

### Conclusion

This study offers important new insights into the workplace experiences of YPSWs. By capturing the perspectives of YPSWs across various settings in the United States, it highlights areas where organizational change and targeted research can advance equity, effectiveness, and sustainability in young adult peer-delivered services. As demand for young adult peer support continues to grow, investing in the conditions that allow YPSWs to thrive will be essential to realizing the full potential of this unique and valuable role.

## Data Availability

Data generated and analysed during this study are available from the authors upon reasonable request.

## References

[CR1] Abraham, K. M., Erickson, P. S., Sata, M. J., & Lewis, S. B. (2021). Job satisfaction and burnout among peer support specialists: The contributions of supervisory mentorship, recovery-oriented workplaces, and role clarity. Advances in Mental Health, 20(1), 38–50. 10.1080/18387357.2021.1977667.

[CR2] Bradley, E. H., Curry, L. A., & Devers, K. J. (2007). Qualitative data analysis for health services research: Developing taxonomy, themes, and theory. *Health Services Research*, *42*(4), 1758–1772. 10.1111/j.1475-6773.2006.00684.x 17286625 10.1111/j.1475-6773.2006.00684.x PMC1955280

[CR3] de Beer, C. R. M., Nooteboom, L. A., van Domburgh, L., de Vreugd, M., Schoones, J. W., & Vermeiren, R. R. J. M. (2022). A systematic review exploring youth peer support for young people with mental health problems. *European Child & Adolescent Psychiatry*, *33*, 2471–2484. 10.1007/s00787-022-02120-5 36495354 10.1007/s00787-022-02120-5 PMC11272732

[CR4] Delman, J., & Klodnick, V. V. (2017). Factors supporting the employment of young adult peer providers: Perspectives of peers and supervisors. *Community Mental Health Journal*, *53*, 811–822. 10.1007/s10597-016-0059-6 27770306 10.1007/s10597-016-0059-6

[CR5] Department of Health and Human Services (2021). *Protecting Youth Mental Health: The U.S. Surgeon General’s Advisory*. https://www.hhs.gov/sites/default/files/surgeon-general-youth-mental-health-advisory.pdf 34982518

[CR6] Donlan, A. E., Lynch, A. D., & Lerner, R. M. (2015). Peer relationships and positive youth development. *Promoting Positive Youth Development: Lessons from the 4-H Study* (pp. 121–136). Springer International Publishing.

[CR7] Firmin, R. L., Mao, S., Bellamy, C. D., & Davidson, L. (2019). Peer support specialists’ experiences of microaggressions. *Psychological Services*, *16*(3), 456–462. 10.1037/ser0000297 30382746 10.1037/ser0000297 PMC6494735

[CR8] Gopalan, G., Lee, S. J., Harris, R., Acri, M. C., & Munson, M. R. (2017). Utilization of peers in services for youth with emotional and behavioral challenges: A scoping review. *Journal of Adolescence*, *55*, 88–115. 10.1016/j.adolescence.2016.12.011 28068538 10.1016/j.adolescence.2016.12.011

[CR9] Halsall, T., Daley, M., Hawke, L. D., Henderson, J., & Matheson, K. (2023). You can create a little bit more closure in your own story when someone really connects with it”: Exploring how involvement in youth peer support work can promote peer development. *International Journal of Mental Health Systems,**17*(1), Article 34. 10.1186/s13033-023-00608-4 37875958 10.1186/s13033-023-00608-4 PMC10594763

[CR10] Harris, P. A., Taylor, R., Thielke, R., Payne, J., Gonzalez, N., & Conde, J. G. (2009). Research electronic data capture (REDCap)-a metadata-driven methodology and workflow process for providing translational research informatics support. *Journal of Biomedical Informatics*, *42*(2), 377–381. 10.1016/j.jbi.2008.08.010 18929686 10.1016/j.jbi.2008.08.010 PMC2700030

[CR11] Harris, P., Taylor, R., Minor, B., Elliot, V., Fernandez, M., & O’Neal, L. (2019). The REDCap consortium: Building an international community of software partners. *Journal of Biomedical Informatics,**95*, Article 103208. 31078660 10.1016/j.jbi.2019.103208 PMC7254481

[CR12] Hermsen-Kritz, M. (2020). *Practice brief: Supporting the youth peer workforce*. Mental Health Technology Transfer Center Network and Research and Training Center for Pathways to Positive Futures.

[CR13] King, S. P., & Bryant, F. B. (2017). The Workplace Intergenerational Climate Scale (WICS): A self-report instrument measuring ageism in the workplace. *Journal of Organizational Behavior,**38*(1), 124–151. 10.1002/job.2118

[CR14] King, M., Dinos, S., Shaw, J., Watson, R., Stevens, S., Passetti, F., & Serfaty, M. (2007). The stigma scale: Development of a standardised measure of the stigma of mental illness. *The British Journal of Psychiatry*, *190*(3), 248–254. 10.1192/bjp.bp.106.024638 17329746 10.1192/bjp.bp.106.024638

[CR15] LeFrançois, B. A. (2014). Adultism. *Encyclopedia of critical psychology* (pp. 47–49). Springer.

[CR16] Noelker, L. S., Ejaz, F. K., Menne, H. L., & Bagaka’s, J. G. (2009). Factors affecting frontline workers’ satisfaction with supervision. *Journal of Aging and Health,**21*(1), 85–101. 10.1177/089826430832864 19144970 10.1177/0898264308328641

[CR17] Ojeda, V. D., Jones, N., Munson, M. R., Berliant, E., & Gilmer, T. P. (2021). Roles of peer specialists and use of mental health services among youth with serious mental illness. *Early Intervention in Psychiatry*, *15*(4), 914–921. 10.1111/eip.13036 32888260 10.1111/eip.13036 PMC9305632

[CR18] Ostrow, L., Cook, J. A., Pelot, M., Salzer, M. S., & Burke-Miller, J. K. (2025). Postcertification wages among certified peer specialists working in peer support and other occupations. *Psychiatric Services*, *76*(4), 350–357. 10.1176/appi.ps.20240195 39696992 10.1176/appi.ps.20240195

[CR19] Scanlan, J. N., Still, M., Radican, J., Henkel, D., Heffernan, T., Farrugia, P., & English, J. (2020). Workplace experiences of mental health consumer peer workers in new South Wales, Australia: A survey study exploring job satisfaction, burnout and turnover intention. *BMC Psychiatry,**20*(1), 270. 10.1186/s12888-020-02688-9 32487169 10.1186/s12888-020-02688-9 PMC7268613

[CR20] Schmidt, S. W. (2004). The Job Training and Job Satisfaction Survey Technical Manual. *Online Submission*.

[CR21] Schmitz, S., Rosa, M. H., Patient, D. L., Vauclair, C. M., & Mariano, J. (2025). Workplace youngism: A scoping review of agism toward younger workers. *Work Aging and Retirement*, waaf004. 10.1093/workar/waaf004

[CR22] Simmons, M. B., Grace, D., Fava, N. J., Coates, D., Dimopoulos-Bick, T., Batchelor, S., & Montague, A. E. (2020). The experiences of youth mental health peer workers over time: A qualitative study with longitudinal analysis. *Community Mental Health Journal*, *56*(5), 906–914. 10.1007/s10597-020-00554-2 31970578 10.1007/s10597-020-00554-2

[CR23] Singh, R., Leung, S., Sawrikar, V., McHugh, C., Hu, N., Ardill-Young, O., Lingam, Raghu, Eapen, Valsamma, Hodgins, Michael, & Curtis, J. (2025). Increasing youth peer workers’ impact through integration: Peer worker perspectives on best practice in youth mental health. *Health Expectations,**28*(2), Article e70223. 10.1111/hex.70223 40098287 10.1111/hex.70223 PMC11913725

[CR24] Tisdale, C., Snowdon, N., Allan, J., Hides, L., Williams, P., & de Andrade, D. (2021). Youth mental health peer support work: A qualitative study exploring the impacts and challenges of operating in a peer support role. *Adolescents*, *1*(4), 400–411. 10.3390/adolescents1040030

[CR25] Torres Stone, R. A., Sabella, K., Lidz, C. W., McKay, C., & Smith, L. M. (2018). The meaning of work for young adults diagnosed with serious mental health conditions. *Psychiatric Rehabilitation Journal*, *41*(4), 290–298. 10.1037/prj0000195 27295134 10.1037/prj0000195

[CR26] University of Illinois-Chicago Center on Integrated Health Care & Self-Directed Recovery (n.d.). National overview of the United States certified peer specialist workforce. https://www.center4healthandsdc.org/map-of-national-peer-training-programs.html

